# Evaluation of nurse-led early graded rehabilitation and pulmonary ultrasound-guided comprehensive chest physical therapy in comatose ICU patients

**DOI:** 10.1097/MD.0000000000047474

**Published:** 2026-01-30

**Authors:** Lili Nie, Chunxiang Chu, Junling Rao, Hongyun Jiang, Xinying He, Shumin Zhang, Fanglan Ye, Jinjuan Li, Guiying Liu, Yuzhen He

**Affiliations:** a Department of Critical Care Medicine, Yue Bei People’s Hospital, Shaoguan City, Guangdong Province, China; b Department of Neonatology, Yue Bei People’s Hospital, Shaoguan City, Guangdong Province, China; c Department of nursing, Yue Bei People’s Hospital, Shaoguan City, Guangdong Province, China

**Keywords:** chest physical therapy, coma, intensive care unit, nurse-led interventions, pulmonary ultrasound, rehabilitation

## Abstract

Patients in the intensive care unit (ICU) who are comatose face respiratory complications and a process of long-term rehabilitation. This study evaluates the combined effect of nurse-led early graded rehabilitation (EGR) and comprehensive chest physical therapy (CCPT) guided by pulmonary ultrasound (PU) on neurological function, respiratory outcomes, and complications in comatose ICU patients. This retrospective study included 120 comatose patients admitted to the ICU from July 2024 to February 2025, divided into a conventional treatment group and a combination treatment group. The latter accepted the CCPT guided by EGR and PU. Evaluate the results using Glasgow Coma Scale, Barthel Index, respiratory parameters, and incidence of complications. The data were analyzed using *t*-test and logistic regression. The combination therapy group demonstrated significant improvements in the Glasgow Coma Scale (6.32 ± 1.15 vs 5.85 ± 1.26; *P* = .035). Respiratory parameters, including higher tidal volumes (239.57 ± 76.07 mL vs194.78 ± 55.34 mL; *P* < .001) and oxygenation indices (245.67 ± 27.51 mm Hg vs 235.34 ± 25.08 mm Hg; *P* = .034), were also enhanced. Significant reductions in pulmonary infection (1.67% vs 15%; *P* = .021) and deep vein thrombosis (1.67% vs 13.33%; *P* = .038) were observed. Integrating EGR with PU-guided CCPT significantly improves neurological function, functional independence, and respiratory outcomes while reducing critical complications in comatose ICU patients. This combined therapeutic strategy represents a highly effective approach to enhancing recovery in critical care settings.

## 1. Introduction

Coma patients in intensive care units (ICUs) represent a particularly challenging cohort due to their critical condition and susceptibility to a myriad of complications.^[1,2]^ These patients typically experience prolonged immobility, mechanical ventilation, and diminished autonomic functions, translating to high morbidity and complex management needs.^[3,4]^ Existing therapeutic strategies focus on the stabilization of vital functions, prevention of secondary brain injury, and mitigation of associated complications such as pneumonia, deep vein thrombosis (DVT), and muscle atrophy.^[5]^ Despite advancements in general ICU care, the effective rehabilitation of comatose patients remains a compelling challenge with a significant impact on patient outcomes and healthcare resources.^[6,7]^

Early rehabilitation has emerged as a crucial intervention in the ICU setting, designed to counteract the adverse effects of prolonged immobility and enhance patient outcomes.^[8,9]^ Nurse-led early graded rehabilitation (EGR) represents a structured approach aiming to incrementally improve a patient’s physical condition through tailored interventions that address individual needs and progress.^[10,11]^ EGR utilizes a stepwise methodology that begins with passive exercises and progresses to active movements as patient consciousness and strength improve.^[12]^ The role of nursing staff in this paradigm is pivotal, as they are integral in providing continuous, patient-centered care, ensuring that interventions are timely and precisely adjusted according to the patient’s response.^[13]^

Pulmonary complications, particularly in comatose patients, threaten respiratory stability and can prolong ICU stays or increase morbidity and mortality.^[14,15]^ Targeted comprehensive chest physical therapy (CCPT) has been recognized for its role in improving lung function, facilitating mucus clearance, and enhancing respiratory mechanics.^[16,17]^ Techniques incorporated in CCPT include manual chest percussion, vibration, postural drainage, and patient positioning.^[18]^ These interventions are designed to maintain pulmonary hygiene and prevent complications such as ventilator-associated pneumonia (VAP).^[19]^

Recent advances in medical imaging, particularly pulmonary ultrasound (PU), have offered enhanced capabilities for real-time monitoring and targeting of therapeutic interventions.^[20]^ PU provides a noninvasive, bedside tool that allows for the dynamic assessment of lung and diaphragmatic function.^[21]^ This real-time feedback is invaluable in guiding CCPT interventions, ensuring that therapeutic actions are both appropriate and effective. While the individual benefits of EGR and CCPT are well documented, their combined application under the guidance of PU has not been extensively researched, particularly in the context of comatose patients. This study aims to investigate the therapeutic efficacy of integrating an EGR nursing plan with targeted CCPT, guided by PU, on coma patients in the ICU.

## 2. Materials and methods

### 2.1. Case selection

This study was approved by the Ethics Committee of Yue Bei People’s Hospital. This retrospective cohort study selected 120 comatose patients admitted to our hospital’s ICU from July 2024 to February 2025 as its subjects. Inclusion criteria: age above 18 years; control of active cerebral hemorrhage has been substantially achieved; the presence of a consciousness disorder (Glasgow Coma Scale [GCS] score below 8) after ICU admission (the diagnosis of coma was made based on the American Congress of Rehabilitation Medicine criteria for coma^[22]^); and coma duration exceeding 10 hours. Exclusion criteria: patients with a “do not resuscitate” order; patients expected to have a persistently disturbed consciousness (e.g., due to anoxic encephalopathy, brainstem hemorrhage, or head trauma); patients with a history of chronic dementia, psychosis, mental retardation, or neuromuscular disease; and patients with visual or hearing impairments.

This study was approved by the Ethics Committee of Yuebei People’s Hospital (YBEC-KY [2023] No. [077], approved on September 19, 2023) in accordance with regulatory and ethical guidelines pertaining to retrospective research studies. Informed consent was waived for this retrospective study due to the exclusive use of de-identified patient data, which posed no potential harm or impact on patient care.

Pulmonary infection was diagnosed based on combined clinical criteria, including fever (>38°C), increased purulent sputum, leukocytosis or elevated inflammatory markers, new or progressive infiltrates on chest imaging, and/or positive sputum or bronchoalveolar lavage cultures. A diagnosis required at least 2 clinical findings plus radiological evidence.

### 2.2. Grouping and treatment methods

In this study, patients were divided into 2 groups based on the treatment methods used: the conventional treatment group and the combined treatment group, with 60 patients in each group. The conventional treatment group received a nurse-led EGR plan designed for severe illness. This plan included position management, lung recruitment through ventilation and manual methods, and peripheral muscle strength retraining, all based on a comprehensive evaluation of the patient’s condition.

**Figure 1.**
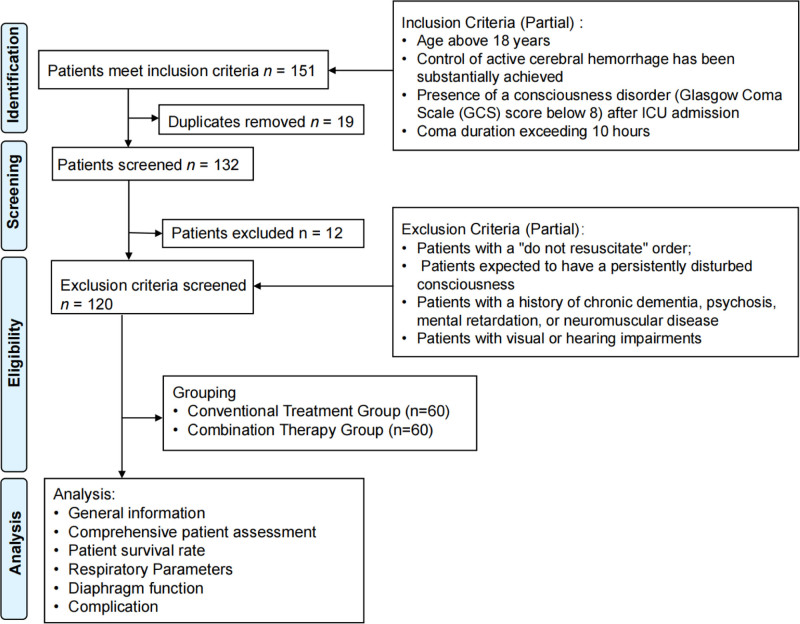
Study design flow chart. ICU = intensive care unit.

In addition to the nurse-led EGR plan, the combined treatment group received further interventions guided by lung ultrasound. These additional treatments involved manual chest tapping and shaking to facilitate mucus drainage, and the application of positive respiratory pressure ranging from 10 to 25 cm H_2_O. This CCPT also included techniques such as bilateral paraspinal suppression, bilateral rib elevation, diaphragmatic fascia release, and soft fascia release at the chest entrance. Both groups underwent the respective interventions for a period of 6 weeks. The study design flow chart is shown in Figure 1. Chest physical therapy maneuvers were delivered twice daily, each session lasting 20 to 25 minutes. Manual percussion and vibration were applied at a frequency of 3 to 5 Hz with moderate intensity tolerated by the patient. Postural drainage was maintained for 5 minutes per position. Positive expiratory pressure was administered at 10 to 25 cm H₂O for 10 minutes, depending on patient tolerance. Diaphragmatic fascia release and rib elevation techniques were performed gently for 5 to 7 minutes each.

### 2.3. Sample size calculation

The sample size for this study was calculated based on previous studies that evaluated the efficacy of early rehabilitation and chest physical therapy in ICU patients. Considering a clinically significant difference in the primary outcome (GCS score improvement) of at least 0.5 points with a standard deviation of 1.2, a power of 80%, and a significance level of 0.05, a minimum of 54 patients per group was required to detect this difference. To account for potential dropouts and missing data, the final sample size was increased to 60 patients per group.

### 2.4. Observation indicators

The primary observation indicators in this study included the GCS, respiratory parameters (tidal volume, oxygenation index, partial pressure of carbon dioxide [PaCO_2_], and respiratory rate), and diaphragm function (diaphragm thickness at end of inhalation and exhalation [DTei and DTee, respectively] and diaphragm thickening fraction [DTF]). These primary indicators were assessed at baseline and then every 2 weeks until the end of the 6-week intervention period by a trained team of nurses and respiratory therapists. Secondary observation indicators included the incidence of complications such as pulmonary infection, DVT, VAP, and barotrauma, which were continuously monitored throughout the intervention period by the ICU medical staff and recorded at the time of occurrence.

### 2.5. Glasgow Coma Scale

The GCS evaluates eye, verbal, and motor responses, combining these to form the Coma Index. For eye response (E), scoring ranges from 4 points for spontaneous eye opening, 3 points for eye opening to verbal commands within 3 minutes, 2 points for response to painful stimuli, and 1 point for no response. Verbal response (V) ranges from 5 points for organized, oriented communication, 4 points for confused responses, 3 points for speech limited to single words, 2 points for incomprehensible sounds, and 1 point for no response; patients unable to speak due to intubation or speech impairment are marked “T” and “D,” respectively. Motor response (M) is scored from 6 points for obeying commands, 5 points for localizing pain, 4 points for withdrawing from pain, 3 points for abnormal flexion, 2 points for abnormal extension, to 1 point for no response. The scale’s reliability is reported as 0.9.^[23]^

### 2.6. Acute Physiology and Chronic Health Evaluation II

The Acute Physiology and Chronic Health Evaluation II (APACHE II) score consists of 3 components: the acute physiological score (ranging from 0–60 points), age score (ranging from 0–6 points), and chronic health score (based on complications and surgical type, ranging from 0–5 points). The acute physiological score is determined by evaluating the most severe (either highest or lowest) physiological readings within the first 24 hours of ICU admission, using the higher of the scores. The age score is categorized into 5 stages, from below 44 years to over 75 years, with points assigned from 0 to 6. The chronic health score assesses the presence of chronic organ dysfunction or immune suppression prior to admission. Patients meeting these criteria score 2 points for scheduled surgeries but receive 5 points for emergency surgeries or nonsurgical admissions.^[24]^

### 2.7. Diaphragm ultrasound

Immediately following the initial clinical examination in the emergency department – provided the patient was not in a critical condition – two ultrasound operators, each with 5 years of experience, conducted ultrasound scans. These operators proceeded to perform a diaphragmatic ultrasound examination on the patient. Utilizing a portable ultrasound machine (TE7S; Mindray Biomedical Electronics Co., Ltd, Shenzhen, China), they employed a 3.5 MHz convex transducer to assess diaphragm excursions in the subcostal view and a 10 MHz linear transducer to measure diaphragm thickness in the zone of apposition. The right hemidiaphragm was measured with the patient in a supine position, as posture can significantly influence diaphragm thickness. All sonographic recordings were saved for subsequent analysis, and each measurement was taken 3 times, with the average value calculated.

### 2.8. Pulmonary ultrasound

We utilized a portable Doppler ultrasound machine (TE7S; Mindray Biomedical Electronics Co., Ltd, Shenzhen, China) equipped with a 10 to 12 MHz linear transducer to measure lung and respiratory parameters. The ultrasonographic examinations were conducted by dividing each hemithorax into 3 distinct regions: the anterior area (bounded by the parasternal and anterior axillary lines), the lateral area (between the anterior and posterior axillary lines), and the posterior area (beyond the posterior axillary line). Both longitudinal and transverse sections were obtained from the anterior, lateral, and posterior chest walls. Patients were positioned semi-recumbently to scan the anterior sections, in lateral decubitus for the lateral thorax, and sitting for the posterior chest wall scans.

### 2.9. Statistical methods

All analyses were conducted using SPSS version 19 (SPSS Inc., Chicago) and the R software package version 3.4.2 (Free Software Foundation, Inc., Boston). Continuous data were tested for normality using the Shapiro–Wilk test. Normally distributed continuous data are presented as mean ± standard deviation and were compared between groups using unpaired *t*-tests. Non-normally distributed continuous data are presented as median (interquartile range) and were compared using the Mann–Whitney *U* test. Categorical data are presented as frequencies and percentages and were compared between groups using the chi-square test or the Fisher’s exact test when appropriate. Statistical significance was set at *P* < .05.

## 3. Results

### 3.1. General data

In this study, the general information of patients in the conventional treatment group (n = 60) and the combination therapy group (n = 60) was compared, with no statistically (Table 1).

**Table 1:** Comparison of general information between 2 groups.

Parameters	Conventional treatment (n = 60)	Combination therapy (n = 60)	*t*/χ^2^	*P* value
Age (yr)	70.12 ± 5.67	71.87 ± 6.21	1.613	.109
Gender (male/female)	30/30	28/32	0.033	.855
BMI (kg/m^2^)	25.38 ± 2.14	24.86 ± 1.92	1.394	.166
Smoking history	14 (23.33%)	12 (20%)	0.049	.825
Drinking history	13 (21.67%)	15 (25%)	0.047	.829
Hypertension	29 (48.33%)	27 (45%)	0.033	.855
Diabetes	18 (30%)	16 (26.67%)	0.041	.839
Hyperlipidemia	27 (45%)	25 (41.67%)	0.034	.854
ICU admission duration (days)	7.3 ± 2.1	6.7 ± 1.8	1.677	.096
Disease types			1.021	.961
Sepsis	9 (15.00%)	10 (16.67%)		
End-stage renal disease	7 (11.67%)	8 (13.33%)		
Severe pneumonia	7 (11.67%)	9 (15.00%)		
Infectious shock	11 (18.33%)	8 (13.33%)		
Acute heart failure	9 (15.00%)	10 (16.67%)		
Other	17 (28.33%)	15 (25%)		

BMI = body mass index, ICU = intensive care unit.

### 3.2. Comprehensive patient assessment

In the study, a comparison of comprehensive patient assessments between the conventional treatment group and the combination therapy group revealed notable differences (Table 2). Patients in the combination therapy group, which involved a nurse-led EGR nursing plan combined with targeted CCPT guided by PU, demonstrated a statistically significant improvement in the GCS scores (6.32 ± 1.15) compared to the conventional treatment group (5.85 ± 1.26; *P* = .035). However, there was no statistically significant difference in the APACHE II scores between the 2 groups (22.10 ± 3.98 vs 21.45 ± 4.56; *P* = .409). These results indicate that the combination therapy may enhance neurological function and daily living activities in coma patients in the ICU without affecting the overall severity of disease as measured by the APACHE II score.

**Table 2:** Comprehensive patient assessment.

Parameters	Conventional treatment (n = 60)	Combination therapy (n = 60)	*t*	*P* value
GCS	5.85 ± 1.26	6.32 ± 1.15	2.132	.035
APACHE II Score	21.45 ± 4.56	22.10 ± 3.98	0.829	.409

APACHE = Acute Physiology and Chronic Health Evaluation, GCS = Glasgow Coma Scale.

### 3.3. Patient survival rate

In examining the survival rates of patients at 1, 3, and 6 months posttreatment, there were no statistically significant differences noted between the conventional treatment group and the combination therapy group (Table 3). At the 1-month mark, survival rates were 90% (54 out of 60) for the conventional treatment group and 95% (57 out of 60) for the combination therapy group (*P* = .488). At 3 months, survival rates decreased to 65% (39 out of 60) in the conventional treatment group and 70% (42 out of 60) in the combination therapy group (*P* = .697). Despite the observed higher survival rates in the combination therapy group at each interval, these differences were not statistically significant.

**Table 3:** Patient survival rate.

Parameters	Conventional treatment (n = 60)	Combination therapy (n = 60)	χ^2^	*P* value
1 mo	54 (90%)	57 (95%)	0.48	.488
3 mo	39 (65%)	42 (70%)	0.152	.697
6 mo	24 (40%)	30 (50%)	0.842	.359

### 3.4. Respiratory parameters

The comparison of respiratory parameters between the conventional treatment group and the combination therapy group revealed several significant findings (Table 4). Patients receiving the combination therapy, which included a nurse-led EGR nursing plan combined with targeted CCPT guided by PU, exhibited a significantly higher tidal volume (239.57 ± 76.07 mL vs 194.78 ± 55.34 mL; *P* < .001) and an improved oxygenation index (245.67 ± 27.51 vs 235.34 ± 25.08; *P* = .034). Additionally, the PaCO_2_ was significantly higher in the combination therapy group (43.21 ± 4.89 mm Hg) compared to the conventional treatment group (41.02 ± 4.75 mm Hg; *P* = .014). Furthermore, the respiratory rate was significantly lower in the combination therapy group (18.68 ± 2.92 bpm) than in the conventional treatment group (19.75 ± 2.85 bpm; *P* = .044). These results suggest that the combination therapy may lead to improved respiratory function in coma patients in the ICU.

**Table 4:** Respiratory parameters.

Parameter	Conventional treatment (n = 60)	Combination therapy (n = 60)	*t*	*P* value
Tidal volume, V_T_ (mL)	194.78 ± 55.34	239.57 ± 76.07	3.688	<.001
Oxygenation index	235.34 ± 25.08	245.67 ± 27.51	2.149	.034
PaCO_2_ (mm Hg)	41.02 ± 4.75	43.21 ± 4.89	2.495	.014
Respiratory rate (bpm)	19.75 ± 2.85	18.68 ± 2.92	2.037	.044

PaCO_2_ = partial pressure of carbon dioxide.

### 3.5. Diaphragm function

The evaluation of diaphragm function and PU scores between the conventional treatment group and the combination therapy group revealed significant improvements in the latter (Table 5). The combination therapy group, which included a nurse-led EGR nursing plan combined with targeted CCPT guided by PU, had a significantly lower lung ultrasound score (16.06 ± 3.81) compared to the conventional treatment group (17.79 ± 3.30; *P* = .009). Additionally, the DTei was significantly greater in the combination therapy group (0.32 ± 0.06) than in the conventional treatment group (0.29 ± 0.07; *P* = .026), as was the DTee (0.26 ± 0.07 vs 0.20 ± 0.08; *P* < .001). Furthermore, the DTF was significantly higher in the combination therapy group (25.24 ± 3.74) compared to the conventional treatment group (22.98 ± 4.12; *P* = .002). These findings suggest that the combination therapy significantly enhances diaphragm function and improves pulmonary assessment scores in coma patients in the ICU.

**Table 5:** Diaphragm function and PU score.

Parameters	Conventional treatment (n = 60)	Combination therapy (n = 60)	*t*	*P* value
PU (LUS) Score	17.79 ± 3.30	16.06 ± 3.81	2.654	.009
DTei	0.29 ± 0.07	0.32 ± 0.06	2.256	.026
DTee	0.20 ± 0.08	0.26 ± 0.07	4.252	<.001
DTF	22.98 ± 4.12	25.24 ± 3.74	3.145	.002

DTee = diaphragm thickness at the end of exhalation, DTei = diaphragm thickness at the end of inhalation, DTF = diaphragm thickening fraction, LUS = lung ultrasound score, PU = pulmonary ultrasound.

### 3.6. Complication

The comparison of complications between the conventional treatment group and the combination therapy group showed significant differences in certain parameters (Table 6). The combination therapy group, which involved a nurse-led EGR nursing plan combined with targeted CCPT guided by PU, exhibited significantly lower rates of pulmonary infection (1.67% vs 15%; *P* = .021) and DVT (1.67% vs 13.33%; *P* = .038) compared to the conventional treatment group. However, no significant differences were observed in the incidence of VAP (11.67% vs 15%; *P* = .788) and barotrauma (78.33% vs 85%; *I* = 0.479) between the 2 groups. These results suggest that while the combination therapy significantly reduces the occurrence of certain complications like pulmonary infection and DVT, it does not significantly affect the rates of VAP and barotrauma in coma patients in the ICU.

**Table 6:** Complications.

Parameters	Conventional treatment (n = 60)	Combination therapy (n = 60)	χ^2^	*P* value
VAP (%)	9 (15%)	7 (11.67%)	0.072	.788
Barotrauma (%)	51 (85%)	47 (78.33%)	0.501	.479
Pulmonary infection	9 (15%)	1 (1.67%)	5.345	.021
DVT	8 (13.33%)	1 (1.67%)	4.324	.038

DVT = deep vein thrombosis, VAP = ventilator-associated pneumonia.

## 4. Discussion

The present study investigates the therapeutic effects of a nurse-led EGR nursing plan combined with targeted CCPT guided by PU on coma patients in the ICU. The critical nature of coma patients in the ICU presents a significant challenge for medical professionals.^[25,26]^ These patients often encounter issues such as respiratory difficulties, decreased mobility, and an increased risk of complications like pneumonia and DVT.^[27]^ Our study has shown that comprehensive interventions guided by PU can significantly improve the health outcomes of these patients.

The combination therapy led to significant improvements in the GCS scores, indicating better neurological function. One possible explanation is that EGR may stimulate neural pathways and may contribute to enhanced neural plasticity, crucial for brain recovery.^[28]^ The physical manipulations and mobilization techniques used during rehabilitation might contribute to increasing cerebral blood flow, thus optimizing brain oxygenation and nutrient delivery. Enhanced cerebral perfusion can aid in neuronal survival and function, which could manifest as improved coma recovery scores.^[29]^ This improvement suggests that the CCPT, when combined with EGR, fosters better physical functionality. Key interventions such as diaphragmatic fascia release, soft fascia release at the chest entrance, and bilateral paraspinal suppression might contribute to enhanced muscle function and better respiratory mechanics. These techniques can foster improved muscular strength and endurance, critical in regaining the ability to perform daily activities. Pulmonary rehabilitation, inclusive of manual chest tapping, shaking to facilitate mucus drainage, and the application of positive respiratory pressure, addresses 2 crucial aspects: respiratory health and mobility.^[30]^ These interventions are beneficial for preventing atrophy and rigidity of the chest muscles, thereby helping patients regain independence more quickly.^[31]^ The ability to perform daily activities contributes significantly to better patient well-being and reduced hospital stays, thereby accelerating the overall recovery process.

A marked improvement in respiratory parameters such as tidal volume, oxygenation index, and PaCO_2_ was observed in the combination therapy group. These findings can be attributed to the direct effect of chest physical therapy on lung mechanics and gas exchange.^[17]^ Techniques such as bilateral rib elevation and diaphragmatic fascia release likely play roles in improving lung elasticity, enhancing ventilatory function, and facilitating mucus clearance.^[32,33]^ PU guidance ensures that these therapeutic interventions are precisely targeted, enhancing their efficacy.^[34,35]^ For instance, the ability to monitor real-time variations in diaphragm thickness and lung ultrasound scores provides immediate feedback on therapy effectiveness, enabling ongoing adjustments.^[36]^ Improved tidal volume and oxygenation indices reflect better lung recruitment, ventilation-perfusion matching, and more effective airway clearance. These mechanisms collectively contribute to optimized respiratory health, which is vital for coma patients, who are often at risk of respiratory failure and associated complications.^[37]^ The study demonstrated significant improvements in diaphragm function in the combination therapy group, indicated by values for the DTei and DTee, and the DTF. Diaphragmatic function is crucial in maintaining effective ventilation, and its impairment can significantly impact respiratory health and recovery in coma patients.^[38]^ The mechanical act of inhalation and exhalation aids in maintaining diaphragmatic muscle tone and functionality. Interventions focusing on diaphragmatic mobility and thickness are likely to enhance the muscle’s contractility and strength, which can mitigate ventilator dependency and foster quicker liberation from mechanical ventilation.

The combination therapy resulted in decreased pulmonary infections and a lower incidence of DVT. This outcome can be ascribed to the multifaceted impact of the targeted CCPT. Effective mucus clearance reduces the risk of bacterial colonization and subsequent pneumonia.^[39]^ Elevation of the ribs and diaphragmatic fascia release are likely to improve drainage and lung field aeration, reducing infection risks.^[40]^ Lower rates of DVT can be attributed to the enhancement of overall patient mobility and blood circulation. Early mobilization and physical manipulation stimulate venous return, reducing the occurrence of stasis-related thrombotic events. By preventing DVT, the combination therapy mitigates 1 of the severe complications often encountered in long-term ICU patients. The synergistic effects observed in this study can be attributed to the integration of multiple targeted interventions. The nurse-led EGR plan ensures a structured and progressive approach to mobilization, tailored to each patient’s condition. Such personalized care dynamically adapts to the patient’s progress, maximizing functional gains and minimizing complicating factors. PU-guided interventions provide real-time, precise therapeutic delivery.^[41]^ This imaging modality allows caregivers to visualize the mechanical responses of the chest wall and diaphragm, facilitating immediate therapy adjustments.^[42]^ This real-time feedback loop can optimize patient outcomes by allowing continuous refinements based on the evolving patient status, thus enhancing recovery prospects and reducing complications.

Despite the promising findings, the study has notable limitations. The sample size, while sufficient to demonstrate statistical significance, may not be large enough to generalize the results broadly. Future studies should consider larger cohorts and multicenter trials to validate these findings further. Another potential limitation is the homogeneous nature of the study population concerning the types of comas included. Future research should explore whether specific subtypes of coma patients benefit differently from these interventions. Investigating the underlying molecular mechanisms, including inflammatory markers and neuroplasticity indices postintervention, could also provide deeper insights into the therapeutic benefits observed.

This study has several limitations. First, it was conducted in a single center, which may limit generalizability. Second, coma patients had heterogeneous etiologies, but subgroup analyses were not performed; therefore, potential differential responses across coma subtypes remain unknown. Third, the follow-up survival outcomes showed no significant between-group differences, which may be due to insufficient sample size to detect long-term effects. Larger, multicenter randomized studies are warranted to confirm these findings.

## 5. Conclusion

In conclusion, our study demonstrates that a combination of nurse-led EGR nursing plans and targeted CCPT guided by PU significantly benefits coma patients in the ICU. Improvements in neurological function, respiratory performance, diaphragm strength, and a reduction in complications underline the utility of this integrated therapeutic approach. By leveraging real-time PU imaging, caregivers can deliver precisely tailored interventions, thereby optimizing patient outcomes.

## Acknowledgments

The authors express their appreciation to staff in Yue Bei People’s Hospital, for their technical assistance.

## Author contributions

**Conceptualization:** Lili Nie, Chunxiang Chu, Junling Rao, Hongyun Jiang, Xinying He, Shumin Zhang, Jinjuan Li, Guiying Liu, Yuzheng He.

**Data curation:** Lili Nie, Chunxiang Chu, Junling Rao, Hongyun Jiang, Xinying He, Jinjuan Li, Guiying Liu, Yuzheng He.

**Formal analysis:** Lili Nie, Chunxiang Chu, Junling Rao, Hongyun Jiang, Xinying He, Shumin Zhang, Jinjuan Li, Guiying Liu, Yuzheng He.

**Funding acquisition:** Yuzheng He.

**Investigation:** Yuzheng He.

**Writing – original draft:** Lili Nie, Chunxiang Chu, Junling Rao, Shumin Zhang, Fanglan Ye, Jinjuan Li, Yuzheng He.

**Writing – review & editing:** Lili Nie, Junling Rao, Shumin Zhang, Fanglan Ye, Jinjuan Li, Yuzheng He.

## Correction

The funding footnote has been added in the online version and it appears as “This study was supported by Project of Shaoguan Health Research Project in 2024 (No. Y24060), Project of Guangdong Medical Research Foundation in 2024 (No. B2024787), Project approved by Guangdong Nurses Association in 2023 (No. gdshsxh2023ms14), Scientific Research Foundation of Guangdong Economic and Health Association in 2024 (No. 2024-WJMF-81), and Scientific Research Foundation of Guangdong Economic and Health Association in 2024 (No. 2024-WJMZ-43)”.
